# A Bio-Hygromorph Fabricated with Fish Swim Bladder Hydrogel and Wood Flour-Filled Polylactic Acid Scaffold by 3D Printing

**DOI:** 10.3390/ma12182896

**Published:** 2019-09-07

**Authors:** Peng Li, Ling Pan, Dexi Liu, Yubo Tao, Sheldon Q. Shi

**Affiliations:** 1 College of Material Science and Engineering, Northeast Forestry University, Harbin, Heilongjiang 150040, China; 2 Department of Mechanical and Energy Engineering, University of North Texas, Denton, TX 76203, USA

**Keywords:** self-morphing, hydrogel, biomimetic, PLA, FDM, 3D printing

## Abstract

Non-powered adaptive systems are attractive in the construction of environment actuators, meteorosensitive architectures, biomedical devices, and soft robotics. Combining hydrophilic materials and anisotropic structures to mimic self-morphing plant structures has been demonstrated as an effective approach to creating artificial hygromorphs. The convenience of 3D printing technologies in shaping programmable complex structures facilitates the imitation of complex anisotropic plant structures. In this research, we constructed a bio-hygromorph using fish swim bladder hydrogel as the hydrophilic material and wood flour-filled polylactic acid (WPLA) scaffold, which was printed with fused deposition modeling (FDM) 3D printing technology (3DP). The environmental benign bio-hygromorph displayed morphing abilities triggered by moisture content changes, as the fish swim bladder hydrogel swelled and shrunk during absorption and desorption cycles. The strain disproportion of the two-layered composite structure in the bio-hygromorph drove the bending deformation. Stress analyses performed with finite element analysis (FEA) also revealed the mechanism behind the moisture content driven morphing of the bio-hygromorph. Notably, the bio-hygromorph exhibited faster response times to moisture absorption than desorption, which may donate actuators’ different attributes in distinct moisture conditions.

## 1. Introduction

A hygromorph is a natural or artificial object whose shape varies with environmental humidity [1]. Hygromorphs are responsive to ambient environmental changes without additional energy [2], which makes them especially desirable for non-powered adaptive systems such as environment actuators [3], meteorosensitive architecture [4,5], biomedical devices [6], and soft robotics [7].

A typical natural hygromorph is the pine cone. Pine cone scales’ reaction to changes in environmental humidity enables the pine cone to open upon drying or close upon wetting [1,8]. Pine cone scales have two layers of hygroscopic tissues embedded with cellulose fibers. These cellulose fibers of the two layers are oriented almost perpendicular each other [2,8]. This peculiar alignment of cellulose fibers and the unique tissue structural properties of pine cone scales are the bases of the pine cone’s morphing abilities [1,8,9,10]. Natural hygromorphs can also be observed in the movement of wheat awns [11] and the opening of plant seed pods [9,11,12].

Inspired by natural hygromorphs, artificial hygromorphs are generally composite structures consisting of two crucial components: hydrophilic materials and anisotropic structures. Hydrophilic materials can shrink in volume due to evaporation when exposed to a dry atmosphere. When immersed in water or exposed to wet atmosphere, hydrophilic materials reabsorb moisture and swell back [2]. The unique anisotropic structure of the hygromorph controls the magnitude and the direction of its deformations. For example, Reyssat and Mahadevan created a bilayer hygromorph by gluing paper, the hydrophilic material, onto a flat strip of polymer [1]. This type of bilayer structure exhibited similar reactions to humidity as pine cones. Le Duigoua and Castro designed a moisture-induced self-shaping biocomposite by hot pressing hydrophilic flax fiber embedded polypropylene films onto fiberless films to create a bilayer structure [13]. Zhao et al. developed self-morphing composites composed of 3D printed fiber scaffolds that were embedded in commercial water swelling rubbers [7]. Erb et al. fabricated a hygroscopic composite with three different fiber orientations to get programmable bend and twisting [14]. Wang et al. presented composite hydrogels with a layered fibrous structure such as bean pods [6]. Above studies show that there are two philosophies to designing new hygromorphs: further research on hydrophilic materials and the exploration of different deformation controlling structures. 

This paper explored creating a bio-hygromorph using fish swim bladder hydrogels as hydrophilic materials and wood flour-filled polylactic acid (WPLA) scaffolds to build anisotropic structures. Fish swim bladder hydrogels are hydrolysates extracted from fish swim bladder, which are used in fields of medicine and food for their high digestibility, thermal stability, and biocompatibility [15]. The WPLA scaffold was fabricated by fused deposition modeling (FDM) 3D printing technology (3DP). WPLA is widely used as bio-based feedstock for FDM for its degradability [16]. In addition, FDM is one of the most commonly used techniques in 3DP [17]. Previous studies also demonstrated that 3DP can use stimuli-responsive materials to create stimuli-responsive structures [18,19]. Compared with existing designs of artificial hygromorphs, the 3D printed bio-hygromorphs with fish swim bladder hydrogel and WPLA scaffold are completely organic and fully biodegradable.

## 2. Materials and Methods

### 2.1. Composite Structure Design of A Bio-Hygromorph

**Figure 1:**
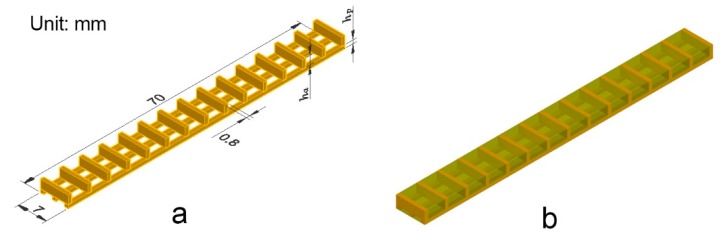
Composite structure design of a bio-hygromorph: (**a**) scaffold model; (**b**) model of the bio-hygromorph with the scaffold embedded in hydrogel.

The bio-hygromorph is composed of a scaffold and fish swim bladder hydrogels. Figure 1a shows the 3D model of the scaffold that was designed with AutoCAD software (Student version 2019). Figure 1b depicts a simulated model of the bio-hygromorph. The bilayer scaffold is formed by crossing a bottom layer of three long vertical stripes with a top layer of fifteen short horizontal stripes at 90°. In this way, the bio-hygromorph is a bilayer composite structure with the bottom layer as the passive layer and the top layer as the active layer. The thicknesses of the passive layer (*h_p_*) and the active layer (*h_a_*) are 0.8 mm and 3.0 mm, respectively.

### 2.2. Preparation of Fish Swim Bladder Hydrolysates

Farmed yellow croaker swim bladders (Larimichthys polyactis) about 5 cm long with water content around 5% were supplied by GaoShunHang Co., Ltd. (Guangdong, China). Enzymatic hydrolysis was performed on the swim bladders using papain (Papain from papaya latex, P-3250, 0.5 units/mg to 2.0 units/mg solid, Sigma-Aldrich, Saint Louis, MO, USA).

The swim bladder was soaked in distilled water for 24 h to remove ash and grease on the surface. After drying and shattering, the swim bladder powder was obtained by screening through a 2 mm mesh sieve. The swim bladder powder was mixed with distilled water at a solid/solvent ratio of 1:20 (w/w). As the solution was stirred continuously, 0.5% papain enzyme was added, and enzymatic hydrolysis of the pulp was performed at 60 °C for 8 h. After full enzyme hydrolysis, the enzyme was killed at 90 °C for 15 min. Next, a filter mesh (90 µm) was used to remove residues. The hydrolysate was dried at 45 °C until the moisture content was 8% and then was ground into particles.

### 2.3. Preparation of 3D Printed Scaffold

The scaffolds were printed using an open source 3D printer (605 S model, Shenzhen Aurora Technology Co, Shenzhen, China). The nozzle diameter of the printer was 0.4 mm, and 1.75 mm WPLA filaments with about 5% wt wood flour (Shenzhen Andsun Co, Shenzhen, China) were used in this study.

The CURA software (3.2.1 version, developed by Ultimaker) was used to set printing parameters. The printing layer height was set to 0.2 mm, the filling density to 100%, the printing speed to 30 mm·s^−1^, the printing temperature to 200 °C, and the hot bed temperature to 60 °C. 

### 2.4. Fabrication of Bio-Hygromorph

Particles of fish swim bladder hydrolysate were weighed and dissolved with deionized water at 70 °C to form an adhesive solution of 30% wt. Aluminum foil was warped around the PLA scaffold to form a mold. The solution was then poured into the mold, burying the scaffold. A bio-hygromorph was obtained after cooling to room temperature and trimming excess hydrogel.

### 2.5. Self-Shaping Test Induced by Moisture Content Changing

In the desorption stage, the bio-hygromorphs were dried under laboratory conditions, i.e., RH = 40% and T = 25 °C. In the absorption stage, the hygromorphs were immersed in deionized water. The weight variation was periodically recorded with a weighing device (10^−4^ g), and pictures of the samples were taken for analyzing the relations between moisture content and curvature variation. 

The moisture content of the sample at recording time *t*, *ϕ_t_*, was determined by Equation (1):

(1) $$\phi_{t}=\left(W_{t}-W_{s}-W_{g}\right)/W_{g}\times 100\%$$

where *W_t_* is the weight of the sample at recording time *t* during the desorption-absorption cycle, *W_g_* is the weight of dry fish swim bladder glue, and *W_s_* is the weight of the scaffold, respectively.

The bending curvature of the sample was estimated by analyzing images using the ImageJ software (version 1.52o). The curvature of samples was measured using the ”fit circle” tool, which draws best fitting circles to estimate the curvature in corresponding images. Three replicas were tested to evaluate the variation of moisture content and bending deformation.

### 2.6. Finite Element Analysis of Bio-Hygromorph Segment

The bio-hygromorph segment models were constructed using the INVENTOR software (Student version 2019). The segment models were used to demonstrate the mechanism of the bending deformation and compare deformation magnitudes of the bio-hygromorph during the desorption-absorption cycles.

**Figure 2:**
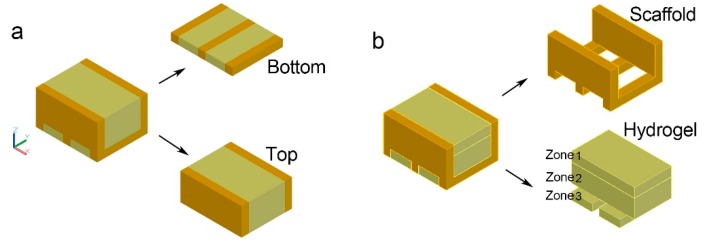
Bio-hygromorph segment model for deformation analysis: (**a**) bilayer structure, with the top layer as the active layer and the bottom layer as the passive layer; (**b**) the hygromorph model contains part models of the scaffold and the hydrogel, with the hydrogel separated into three zones based on possible moisture content. Zones 1 and 3 share identical mechanical properties but are distinct from zone 2.

As shown in Figure 2a, the bio-hygromorph segment model was separated into a bilayer structure to simulate its deformation under the same pressure. Different bending magnitude analyses were performed on one segment of the bio-hygromorph during the desorption-absorption cycle, as shown in Figure 2b. The fish swim bladder hydrogel was separated into three zones to simulate the potential nonuniform distribution of moisture content along its center and its edges during desorption-absorption cycles. Deformation simulations were completed using a stress analysis [finite element analysis (FEA)] tool in INVENTOR software. Materials and constraints were first applied to the model. Young’s modulus: 2.24 GPa for WPLA, 0.124 GPa, and 0.091 GPa for different hydrogel zones [17,20] were used in simulation. The model was then meshed before the force simulation. Rigid surface constraints were applied under the models accordingly. The displacement of the loaded surface of the segment model was calculated.

## 3. Results and Discussion 

### 3.1. Shape Changing of Bio-Hygromorph Responds to Moisture Content

**Figure 3:**
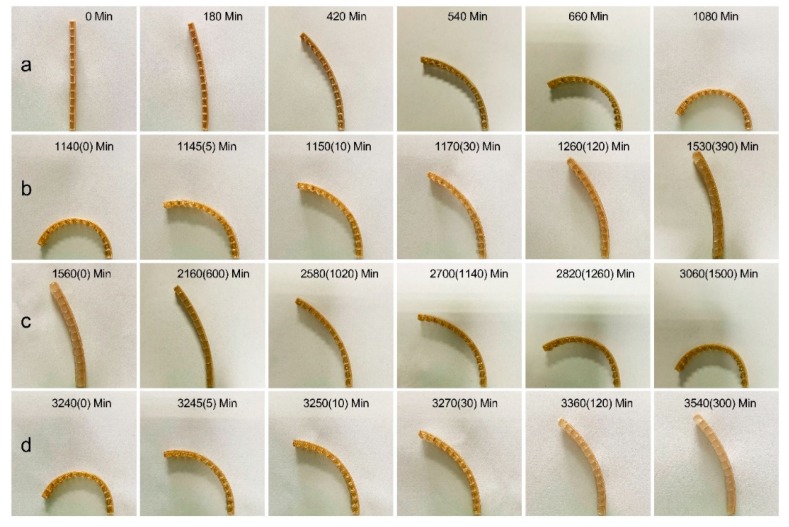
The bio-hygromorph self-shaping during the desorption-absorption cycle: (**a**) bending during the desorption stage of the first cycle; (**b**) straightening during the absorption stage of the first cycle; (**c**,**d**) are deformations during the desorption and the absorption stages of the second cycle, respectively.

Figure 3 presents the typical evolution of the shape-changing bio-hygromorph during the desorption-absorption cycle, exhibiting bending during drying (desorption) and straightening during immersion (absorption). Figure 3a shows the desorption stage of the first cycle. The bio-hygromorph exhibited greater bending towards the active layer during desorption with the progression of time as the fish swim bladder hydrogel dried and hardened. Figure 3b shows the absorption stage of the first cycle. During absorption, the curved bio-hygromorph gradually straightened, though it was unable to completely revert to its original, uncurving state. Figure 3c,d illustrate the desorption stage and the absorption stage of the second cycle, where the bio-hygromorph exhibited bending characteristics similar to that of Figure 3a,b.

**Figure 4:**
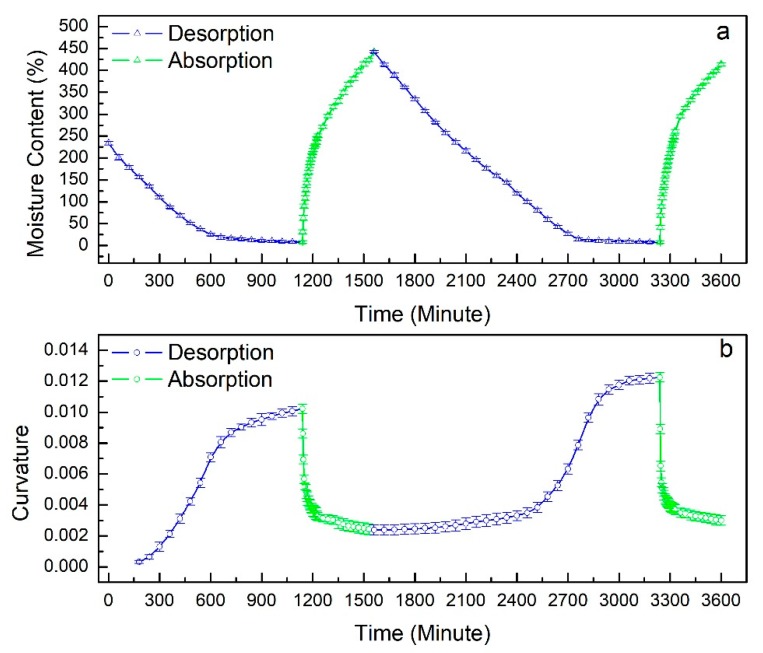
Moisture content and bending curvature evolution during desorption-absorption cycles: (**a**) variation of moisture content during two desorption-absorption cycles; (**b**) variation of curvature during two desorption-absorption cycles.

Figure 4 demonstrates the variations of curvature and moisture content during the desorption-absorption cycle. During the first desorption stage, bending curvature and moisture content behaved as two-step functions of time, i.e., a rapid increase of curvature and a decrease of moisture content followed by a plateau, which was similar to the findings of Le Duigou and Castro [13]. Bending occurred around a moisture content of 170 ± 4.0%. The rate of bending slowed drastically after moisture content fell below 37 ± 2.3%. A similar two-step behavior was observed during the absorption stage. As shown in Figure 4, the absorption stage was far less time consuming than the desorption stage. For example, during desorption, it took 700 min for the bending curvature to increase from 0.003 to 0.01, while the same variation in bending curvature was reverted under 30 min during absorption.

During the second desorption stage, the bending curvature followed a three-step behavior. The moisture content decreased linearly from around 400% to 140 ± 3.4% as the bending curvature increased gradually. Similar to the first cycle, the bending accelerated as moisture content fell below 140 ± 3.4% and decelerated as moisture content fell below 27 ± 3.9%. The behaviors of moisture content and bending curvature exhibited during the second absorption stage were similar to those of the first absorption stage. Initial curvature recovery demonstrated the bio-hygromorphs’ ability to undergo reversible bending actuation.

### 3.2. Moisture Content Driven Bending Principles of Bio-Hygromorph

Based on the principles behind bimetallic thermostats [1,21], assuming that the thicknesses of the constituent passive and active layers were $h_{p}$ and $h_{a}$ with a total thickness $(h=h_{p}+h_{a}$), their Young’s moduli $E_{p}$ and $E_{a}$ and the linear hygrometric expansion coefficients $\alpha_{p}$ and $\alpha_{a}$ were assumed to be independent of the moisture content ∅ of the bio-hygromorph. Under a change of Δ∅, the strains of active layer and passive layer could be expressed as Equations (2) and (3), respectively.

(2) $$\varepsilon_{a}=\alpha_{a}\Delta \emptyset$$

(3) $$\varepsilon_{p}=\alpha_{p}\Delta \emptyset$$

In the absence of external forces, all forces acting on any cross section of the active and the passive layers must be in equilibrium, see Equation (4):

(4) $$F_{p}=-F_{a}=F$$

and torque balance requires Equation (5):

(5) $$\frac{Fh}{2}=M_{p}+M_{a}$$

where $F_{p}$ and $F_{a}$ are the axial forces. If $M_{p}$ and $M_{a}$ are the bending moments in each of the layers, the flexural rigidities (per unit width) of both layers are $E_{p}I_{p}\;$= the flexural rigidity of the passive layer and $E_{a}I_{a}$ = the flexural rigidity of the active layer.

Then, letting *r* = radius of curvature of bio-hygromorph and *k* = curvature of bio-hygromorph, $M_{p}$ and $M_{a}$ could be described as Equations (6) and (7).

(6) $$M_{p}=\frac{E_{p}I_{p}}{r}=k\left(E_{p}I_{p}\right)$$

(7) $$M_{a}=\frac{E_{a}I_{a}}{r}=k\left(E_{a}I_{a}\right)$$

In Equations (6) and (7), $I_{p}$ and $I_{a}$ are the moments of inertia (per unit width), which are associated with $h_{p}$ and $h_{a}$, see Equations (8) and (9):

(8) $$I_{p}=\frac{h_{p}^{3}}{12}$$

(9) $$I_{a}=\frac{h_{a}^{3}}{12}$$

Combining Equations (5)–(9) gives Equation (10):

(10) $$\frac{Fh}{2}=k\left(\frac{E_{p}h_{p}^{3}}{12}+\frac{E_{a}h_{a}^{3}}{12}\right)$$

Finally, the displacement of both layers must be equal at the contact surface of both layers. Thus, see Equation (11):

(11) $$\varepsilon_{p}+\frac{F_{p}}{E_{p}h_{p}}+k\frac{h_{p}}{2}=\varepsilon_{a}+\frac{F_{a}}{E_{a}h_{a}}-k\frac{h_{a}}{2}$$

Combining Equations (9) and (10) yields the change in curvature Equation (12):

(12) $$k=\frac{\left(\varepsilon_{a}-\varepsilon_{p}\right)f\left(m,n\right)}{h}$$

where in Equation (13) [1,2,7,13,21,22]:

(13) $$f\left(m,n\right)=\frac{6{\left(1+m\right)}^{2}}{3{\left(1+m\right)}^{2}+\left(1+mn\right)\left(m^{2}+1/mn\right)}\;$$

and $m=h_{p}/h_{a}$, $n=E_{p}/E_{a}$.

Because $\varepsilon_{a}-\varepsilon_{p}\neq 0$, the differential expansion of both layers results in the bending of the bio-hygromorph.

**Figure 5:**
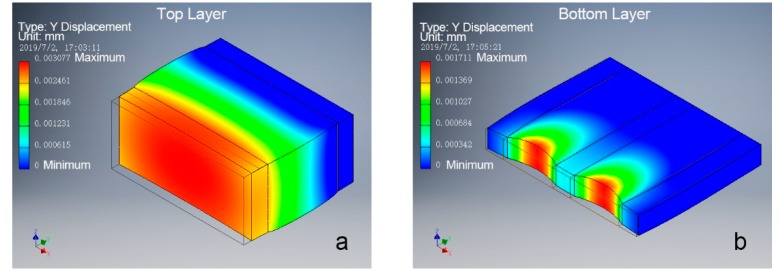
Deformation simulations on a segment model of the bio-hygromorph under identical pressure: (**a**) deformation of the top layer as the active layer; (**b**) deformation of the bottom layer as the passive layer. The active layer underwent greater deformation than the passive layer.

Stress analyses performed on models of the active and the passive layers (Young’s modulus: 2.24 GPa for WPLA, 0.124 GPa for hydrogel) showed similar results, as theorized. Figure 5 illustrates the simulated deformations of the active and the passive layers under the same pressure. As shown in Figure 5, the active layer’s loaded surface underwent greater maximum displacement (Figure 5a) along the direction of force than the passive layer’s (Figure 5b). Thus, $\varepsilon_{a}-\varepsilon_{p}\neq 0$. As a result, the hygromorph underwent deformation and bend.

### 3.3. Relationship between Moisture Content and Curvature of Bio-Hygromorph

**Figure 6:**
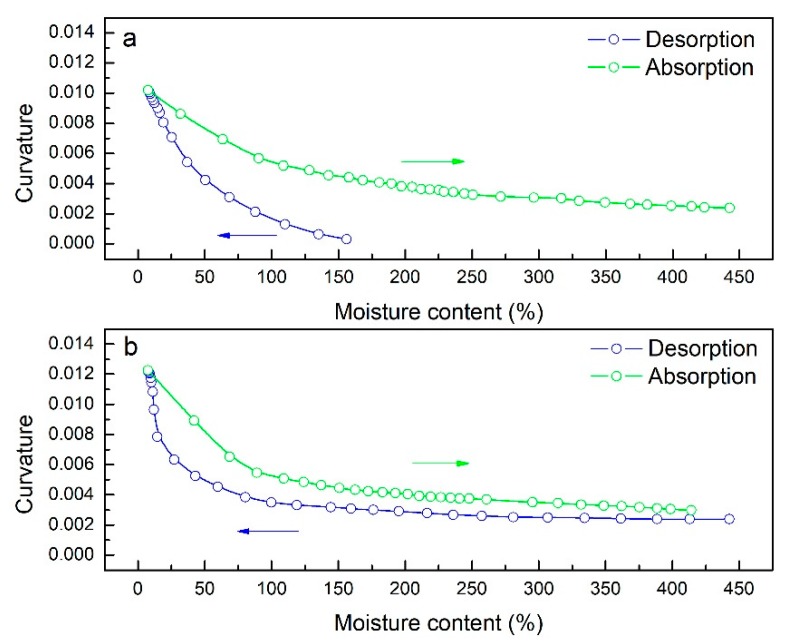
Plots of bending curvature against moisture content. Under identical moisture contents, corresponding bending curvature was consistently greater during absorption than desorption: (**a**) during the first desorption-absorption cycle; (**b**) during the second cycle.

Figure 6a,b shows the relation between moisture content and bending curvature for the first and the second desorption-absorption cycles, respectively. As shown in Figure 6, curvature varied non-linearly with moisture content. As moisture content decreased, bending curvature increased. Notably, significant bending deformation occurred in the moisture content range of 10–160% for desorption and 10–120% for absorption during both cycles.

**Figure 7:**
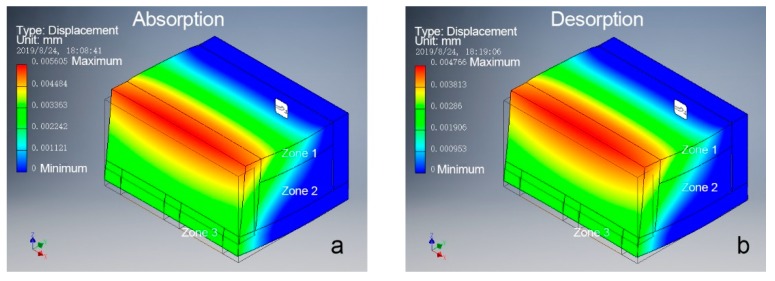
Deformation simulation of a segment model of the bio-hygromorph with the same conditions, with greater deformation for the absorption model than the desorption model: (**a**) deformation during absorption, marked by higher mechanical properties for zone 2 and lower mechanical properties for zones 1 and 3; (**b**) deformation during absorption, marked by lower mechanical properties for zone 2 and higher mechanical properties for zones 1 and 3.

In addition, bending curvature was consistently greater during absorption than desorption for the same moisture content. To investigate the reason behind this phenomenon, bending magnitude analysis was performed on one segment of the bio-hygromorph. The segment was composed of a WPLA scaffold and fish swim bladder hydrogel (Young’s modulus: 2.24 GPa for WPLA, 0.124 GPa and 0.091 GPa for different hydrogel zones), as shown in Figure 2b. The fish swim bladder hydrogel was separated into three zones to simulate the potential nonuniform distribution of moisture content along its center and its edges during the desorption-absorption cycles. Acting as edges, zone 1 and zone 3 shared the same mechanical properties. For absorption, zone 2’s mechanical properties were lower than those of zone 1 and zone 3. For desorption, conversely, zone 2’s mechanical properties were higher. As shown in Figure 7, the simulation results (maximum of displacement) revealed that greater deformations occurred during absorption than desorption, which could be used to explain consistently greater bending curvature during absorption than desorption for the same moisture content.

## 4. Conclusions

The following conclusions can be drawn from this study:

(1) A bio-mimicking, water-driven bio-hygromorph was successfully constructed through embedding a 3D printed WPLA scaffold in fish swim bladder hydrogel.

(2) Pine cone movements can be effectively mimicked by the two-layered composite structure architected in the research. 

(3) The absorption stage is far less time consuming than the desorption stage, and the bio-hygromorph can straighten faster than bend. 

(4) The active component is recyclable and known to be environmentally friendly, which is in line with the expectations of bioinspiration and biomimetics. 

(5) Several avenues of improvement are still available and should be further investigated: hydrolysate content of hydrogel, fiber orientation in scaffold, and scaffold architecture on deformation categories, magnitude, and recovery.
